# Mental health impacts of earthquake on Afghans amidst humanitarian crisis

**DOI:** 10.1016/j.amsu.2022.104521

**Published:** 2022-09-01

**Authors:** Sayed Jawad Hussaini, Syed Hasham Ali, Zainab Syyeda Rahmat, Zarmina Islam, Zoaib Habib Tharwani

**Affiliations:** Faculty of Medicine, Dow Medical College, Dow University of Health Science, Pakistan

**Keywords:** Mental health, Earthquake, Humanitarian crisis, Afghanistan, WHO, World Health Organization, NGOs, Non-Governmental Organizations, HCWs, Health Care Workers, PTSD, Post-Traumatic Stress Disorder, MDD, Major Depressive Disorder

## Abstract

The deadly earthquake in southeast Afghanistan on June 22, 2022 was a tragedy amidst the country's humanitarian crisis. It cost more than a thousand people's lives, caused three times more injuries, and destroyed many houses, mainly in Spera, Giyan, and Barmal districts. WHO and other NGOs responded to incidence, focusing on physical injuries, food shortage, and shelter, while mental health was not adequately emphasized. Almost half of the Afghanistan population suffers from mental disorders due to decades of civil war, economic instability, and natural disasters. The recent earthquake further exacerbates the mental conditions among earthquake victims and their families, making them vulnerable to severe mental disorders. The absence of local mental facilities and proper roads delayed the early response and made the follow-up difficult leading to serious mental issues and costly management. Although WHO and HealthNet TPO sent their mental health professionals to consult the earthquake victims and train health care workers, the sociocultural beliefs made the approach difficult and its result suspicious. Moreover, the stigma around mental health and the lack of female HCWs stop people from seeking mental healthcare.

## 1 Introduction

Mental health is an integral part of health that is essential for personal, community, and socioeconomic development; exposure to unfavourable social, economic, political, and environmental circumstances such as poverty, violence, inequity, and natural disasters, increase the risk for mental disorders [1]. Afghan people have suffered four decades of civil war, an unstable economy, and frequent natural disasters leading to a high rate of mental conditions; about 47% of the population is affected by mental illnesses [2]. Besides, the stigma associated with mental health hinders access to available mental care, especially in rural areas with low education [3]. The recent 5.9 magnitude earthquake on 22 June caused 1039 deaths, 2949 injuries, and 4500 house damage, mostly in Giyan, Barmal, and Ziruk districts in Paktika province and Spera in Khost province, further exacerbating the mental health of the country [4]. On top of that, the poor availability of mental health services has made it difficult to cope with traumatic experiences such as earthquakes. Moreover, 79.3% of health expenses that were covered by the foreign fund were stopped after the Taliban took over the country in august 2021 [5,6]. Stigma compounded by socioeconomic disparities has created a complex trajectory for assisting those with mental health conditions.

Although literature exists on the state of mental health in Afghanistan before the COVID-19 pandemic, it is very sparse after the pandemic, especially within the context of the recent earthquake. Therefore, this paper discusses the recent earthquake's impact on Afghans' mental health and recommendations to address this.

## 2 Impact of earthquake on mental health

Earthquakes increase the prevalence of mental health disorders such as depression, anxiety, post-traumatic stress disorder (PTSD), sleep disorder, and substance abuse [7]. Psychological response to a disaster manifests in 5 stages; warning, impact, inventory, short-term, and long-term recovery; response to earthquakes begins in the impact stage leading to severe reactions [8]. Afghanistan located in the mountainous Hindu Kush region, part of the Aliped belt, is the second most tectonically active region in the world; in addition, the collision of Indian and Eurasian tectonic plates near Afghanistan's border with Pakistan made the country prone to earthquakes and its associated mental conditions [9]. World Health Organization stated that the incidence of mental conditions after an emergency is more than double the general population in a conflict-affected area. The point prevalence for affected adult is 22% with mild to moderate depression, anxiety, and PTSD accounting for 17% and severe depression/anxiety/PTSD, schizophrenia, and bipolar disorder for 5% [10]. Decades of conflict between the government and Taliban held Afghans defenceless against mental disorders. According to a 2020 survey that interviewed 4433 people from 8 provinces of Afghanistan, the prevalence of mental health issues is 47.12%, with impaired function due to mental conditions at 39.44% [2]. The recent earthquake in southeast Afghanistan exposed the rural population to extreme health issues, especially mental disorders; physical trauma, loss of a family member/loved ones, loss of housing, financial loss, and displacement, female sex, low education defined as the risk factors [7,11]. The absence of local mental care, natural barriers, and improper roads hinder the early response and increase the risk of long-term impacts on society which may cause society dysfunction, leading to increased family conflicts, violence, aggression, suicide thoughts, and substance abuse [4,8].

## 3 Challenges and implications

The administration of mental healthcare has faced many challenges in the country and was further defied by the recent earthquake. The main challenge is poverty, with the latest International Wealth Index data showing that 99.7% of the families in Paktika are poor [12]. The earthquake demolished many houses, displacing families with no budget to rebuild their homes, potentially contributing to a sense of loss, anxiety, post-traumatic stress disorder, and major depressive disorder [13]. Mental health professionals could treat these mental conditions if not for the poor accessibility of the region and lack of mental healthcare facilities [14]. The tarmac roads are inadequate in Paktika, and the population is forced to use dirt tracks passing through mountainous terrain, with the earthquake causing ground displacement and surface rupture, making the dirt roads useless hence delaying access to health facilities [15]. Earthquakes can also increase the fatalistic beliefs in society, with many Muslims looking to the event as a Musibah or trial from God, which can be a psychological hindrance to seeking mental health care [16]. Ignorance of the potential rise in mental disorders causes many emergent societal issues. Afghanistan has suffered massive drug abuse due to decades of conflict, fueled by poor mental health [17]. The increased PTSD and MDD may deepen the problem by pushing more people into narcotics; a bidirectional link has been shown between patients suffering from PTSD or MDD and drug abuse [18].

Moreover, according to a 2021 study, MDD patients are significantly associated with aggressive bursts [19]. In Afghanistan, where most of the population lives in joint families, this can lead to family conflicts [20]. Perpetuating a vicious cycle where lack of mental healthcare for victims will lead to drug abuse and violence, worsening overall mental health conditions and increasing the burden on existing mental care facilities, indicating that government and aiding NGOs must consider mental health responses early and for the long run.

## 4 Efforts and recommendations

Fortunately, multiple organizations have responded to the urgent need for mental health facilities in Afghanistan. HealthNet TPO is a Dutch organization created by Doctors without Borders and has provided services in Afghanistan since 1994. The organization has focused on assimilating mental health services into healthcare facilities in Afghanistan since 2005 [21]. Training offered by HealthNet TPO has allowed 325 Afghans to become psychosocial specialists, providing support to civilians within the health facilities. In addition, the organization has trained over 200 doctors, midwives, and nurses in mental healthcare and has established 56 mental health facilities throughout the country.

When the deadly earthquake hit, HealthNet TPO responded by providing mobile health teams to the affected areas, especially in the province of Khost. Since it ran health facilities in this province, medical staff were able to prepare a 50-bed emergency location for the first casualties, as stated by Willem Reusing, Director of Operations [22]. Three mobile teams were quickly sent to another affected area of Spera. Humanitarian agencies such as Islamic Relief were quick to provide emergency aid but have expressed the overwhelming strain on agencies to support people in need [23].

While these organizations provide vital emergency relief, mental health consultation is inadequate. Along with support from other global agencies, the intercalation of religion with mental health will reduce psychological therapy's stigma and promote seeking consultation. Notably, the removal of international restrictions on emergency aid, both medical and psychiatric, is imminent to alleviate this growing mental health burden. While allowing aid to pour into the country, this will also allow patients to receive psychiatric care in neighbouring countries, where facilities are better equipped.

Mental healthcare services have been proven to reduce the risk of chronic diseases related to inadequate consultation, such as anxiety, stress, and substance abuse [24]. Therefore, countries around the globe, as well as NGOs, must play a more active role in providing mental healthcare services in Afghanistan, especially since the civilians are under extreme mental anguish as 1 in 2 Afghans suffer from psychological stress [25]. The effect of devastated infrastructure may be mitigated by the increased use of telemedicine in collaboration with Afghan Wireless, Afghanistan's largest telecom provider, which would allow remote regions mental health support with the added benefit of some measure of anonymity as technology such as voice filters allow people to communicate better while concealing their identity. Prevalent conservative attitudes restrict women's ability to seek counselling; therefore, it would help if counselling teams were more gender-balanced, facilitating a woman-to-woman interaction deemed acceptable by the rural Afghan populace. Camps established to aid victims should incorporate recreational activities to engage the youths' energies, a strategy is previously shown to improve mental health [26]. Employment, while also being a source of monetary income, can aid the mental health of many victims, particularly women. Activities like embroidery or tailoring should be embedded into a comprehensive rehabilitation strategy [27]. The efficacy of such steps depends on honest dialogue with the ruling government and the influential clergy. Their endorsement of such causes may make the Afghan population much more receptive to seeking mental health aid.

## 5 Conclusion

Afghans have suffered from mental health issues for multiple decades; however, the recent earthquake has further exacerbated the concerns about mental health. Considering the remoteness of the region, current humanitarian crisis, and social stigma associated with mental health, it is challenging to respond to mental health needs. International organizations should invest more in mental health services and train enough health care workers to address the needs of Afghans through creative initiatives and rehabilitation strategies. Besides, people's awareness about mental health should be raised to eliminate the stigma.

## Ethics approval and consent to participate

Not applicable.

## Sources of funding

None.

## Authors’ contributions

Sayed Jawad Hussaini conceived the idea, came up with the design, wrote the abstract, introduction, impact of earthquake on mental health, conclusion, and edited the revised draft and organized references; Syed Hasham Ali wrote the challenges and implications, Zainab Syyeda Rahmat wrote the Efforts and recommendations, Zarmina Islam made the critical comments and revision, Zoaib Habib Tharwani revised the last draft and guide with submission process.

## Consent for publication

All authors agreed to the publication of this manuscript.

## Guarantor

N/A.

## Declaration of competing interest

The authors declare that they have no competing interests.
